# Sponge Long Non-Coding RNAs Are Expressed in Specific Cell Types and Conserved Networks

**DOI:** 10.3390/ncrna4010006

**Published:** 2018-03-07

**Authors:** Federico Gaiti, William L. Hatleberg, Miloš Tanurdžić, Bernard M. Degnan

**Affiliations:** 1 School of Biological Sciences, University of Queensland, Brisbane, QLD 4072, Australia; feg2007@med.cornell.edu (F.G.); williamhatleberg@gmail.com (W.L.H.); m.tanurdzic@uq.edu.au (M.T.); 2 Department of Medicine, Weill Cornell Medicine, and New York Genome Center, New York, NY 10021, USA; 3 Department of Biological Sciences, Carnegie Mellon University, Pittsburgh, PA 15213, USA

**Keywords:** evolution, cell type, gene expression, complexity, animals, long non-coding RNAs, development

## Abstract

Although developmental regulation by long non-coding RNAs (lncRNAs) appears to be a widespread feature amongst animals, the origin and level of evolutionary conservation of this mode of regulation remain unclear. We have previously demonstrated that the sponge *Amphimedon queenslandica*—a morphologically-simple animal—developmentally expresses an array of lncRNAs in manner akin to more complex bilaterians (insects + vertebrates). Here, we first show that *Amphimedon* lncRNAs are expressed in specific cell types in larvae, juveniles and adults. Thus, as in bilaterians, sponge developmental regulation involves the dynamic, cell type- and context-specific regulation of specific lncRNAs. Second, by comparing gene co-expression networks between *Amphimedon queenslandica* and *Sycon ciliatum*—a distantly-related calcisponge—we identify several putative co-expression modules that appear to be shared in sponges; these network-embedded sponge lncRNAs have no discernable sequence similarity. Together, these results suggest sponge lncRNAs are developmentally regulated and operate in conserved gene regulatory networks, as appears to be the case in more complex bilaterians.

## 1. Introduction

Animal genomes encode thousands of long non-coding RNAs (lncRNAs) with no apparent protein coding capacity [1,2,3,4,5,6,7,8,9,10,11,12,13,14]. Despite their similarities with protein-coding genes, such as being spliced, transcribed by RNA polymerase II, and possessing 5′-terminal methylguanosine caps and poly(A) 3′-tails [15,16], lncRNAs tend to be expressed in specific cell types [17] and in a more tissue- and developmental stage-restricted manner than protein-coding genes [2,18,19,20,21,22,23,24]. This suggests that animal development requires the fine-scale regulation of expression of specific lncRNAs [25].

However, lncRNAs are rapidly evolving and exhibit poor primary sequence similarity between species. Orthologues are difficult to identify [26], thus precluding a detailed understanding of their evolution in terms of sequence, structure and function. In fact, while the role of lncRNAs in the regulation of developmental gene activity appears to be widespread amongst animals [18,20,22,27,28,29,30,31,32,33,34,35,36,37,38,39,40,41], only a handful of lncRNAs have thus far been shown to possess conserved function(s) in evolutionarily divergent animals, with all functional studies to date being restricted to bilaterians [36,39,42,43,44].

**Figure 1:**
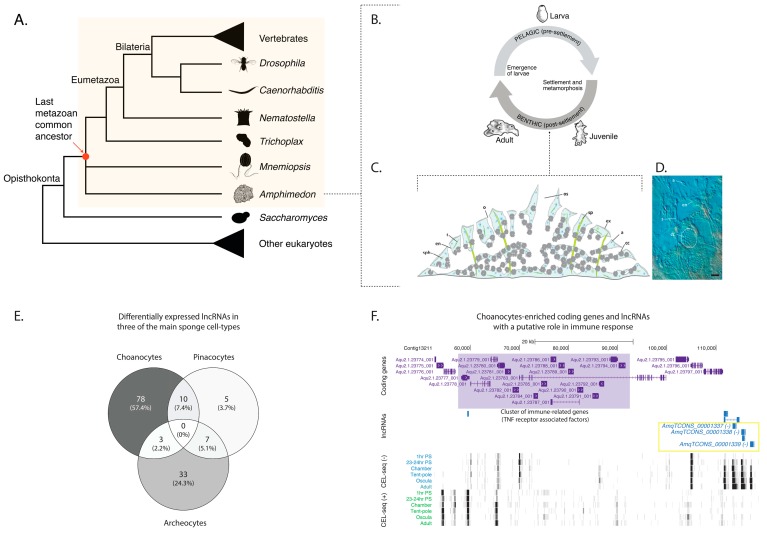
*Amphimedon queenslandica* long non-coding RNAs (lncRNAs) are enriched in specific cell types. (**A**) Phylogenetic tree of selected species with well-described genomes. Yellow background highlights the animal kingdom. The position of *Amphimedon* queenslandica and *Mnemiopsis leidyi* is indicated as a polytomy given the current debate on the branching order of poriferan and ctenophore lineages [52,53]; (**B**) Schematic representation of *Amphimedon queenslandica* life cycle. Larvae (oval shaped, 400–600 µm long) emerge from maternal brood chambers and then swim in the water column before they develop competence to settle and initiate metamorphosis into a juvenile. The juvenile body plan, which displays the hallmarks of the adult body plan, including an aquiferous system with canals, choanocytes chambers, and oscula, is the outcome of the dramatic reorganization of the radially-symmetrical, bi- or trilayered larva. This juvenile will then grow and mature into a benthic adult (ranging from 10–30 cm^3^) [51]; (**C**) Diagram of a juvenile sponge body plan. Water flows into the internal aquiferous system via the ostium and out via the osculum. The mesohyl is shown in blue and populated by archeocytes and other cell types, including sclerocytes and spherulous cells. Adapted from [49]; (**D**) Optical section of a 3-day-old *Amphimedon queenslandica* juvenile showing internal morphology and some cell types. Archeocyte (a), choanocyte chamber (cc), endopinacoderm (en), exopinacoderm (ex), ostium (o), osculum (os), sclerocyte (s), spicule (sp), and spherulous cell (sph). Scale bar: 10 μm. Adapted from [49]; (**E**) Venn diagram denoting the proportion of differentially expressed lncRNAs detected in each of the three cell-type specific transcriptome datasets; (**F**) Subset of *Amphimedon* lncRNAs (*AmqTCONS_00001337-9*) are in close genomic proximity to a cluster of immune-related genes. Coding genes (purple) and long non-coding RNAs (blue) are shown, along with signal coverage tracks showing CEL-seq expression. A grey scale indicates CEL-seq (Cell Expression by Linear amplification and sequencing) expression level: white (no-expression); black (highest expression). The shaded purple area represents the cluster of immune-related genes [Tnf receptor-associated factors (TRAFs)]. Figure was generated using a local instance of the UCSC genome browser [54].

Porifera (sponges) are one of the oldest surviving phyletic lineages of animals, diverging from other animals around 700 Mya [45]. As such, they are an informative lineage for understanding the origin and evolution of animal lncRNAs. Traits shared between sponges and all other animals likely reflect shared inheritance from their last common ancestor (Figure 1A). Sponges consist of four classes—Demospongiae, Calcarea, Homoscleromorpha and Hexactinellida [46,47,48]—of morphologically-simple animals (they lack true gut, nerves and muscles) that share a common body organization and appear to have diverged from each other over 650 Mya [45] (Figure S1). They possess an internal network of canals and ciliated choanocyte chambers lined with epithelial cells, primarily endopinacocytes and choanocytes, and are separated from the external environment by another epithelial layer, the exopinacoderm. Choanocyte chambers pump water through this internal aquiferous canal system, drawing food into the sponge. Between the internal and external epithelial layers is the collagenous mesohyl, which is enriched with multiple types of amoebocytes, including the pluripotent stem cell type—the archeocyte. This juvenile body plan is the outcome of the dramatic reorganization of the radially-symmetrical, bi- or trilayered larva at metamorphosis [49,50,51] (Figure 1B–D).

Despite being one of the simplest animals, sponges developmentally express an array of lncRNAs in manner akin to more morphologically-complex insects and vertebrates [18,20]. These lncRNAs also appear to belong to co-expressed developmental gene modules [18,20], suggesting that complex genome regulation by lncRNAs is an ancient animal trait. However, whether sponge lncRNAs possess other features conserved in bilaterian lncRNAs, including cell type-restricted expression, remains unclear. Here, following our identification and characterization of lncRNAs in the demosponge *Amphimedon queenslandica* (herein *Amphimedon*) [20], we analyze spatially- and cell type-restricted expression patterns of a subset of *Amphimedon* lncRNAs. We also identify a number of modules of co-expressed homologous protein-coding genes and lncRNAs that appear to be conserved between *Amphimedon* and the calcareous sponge *Sycon ciliatum* (herein *Sycon*) [18,20]; these sponges diverged from each other more than 650 Mya [45]. Together, these analyses of sponge lncRNAs further implicate this class of rapidly-evolving non-coding RNAs in the regulation of metazoan development.

## 2. Results and Discussion

### 2.1. Sponge lncRNAs Are Enriched in Specific Cell Types

Long non-coding RNAs exhibit cell type-specific restricted expression patterns in bilaterians [2,17,19,21,24,55,56,57]. To assess whether this is the case for *Amphimedon* lncRNAs and to better understand their putative role(s) in a cellular context, we analyzed cell-type specific CEL-seq (Cell Expression by Linear amplification and sequencing) datasets from three of the main cell-types of sponges–archeocytes, pinacocytes, and choanocytes (Figure 1C,D) [58]. Reads from these datasets were mapped to *Amphimedon* lncRNA collection (*n* = 2935) [20] to identify cell-type specific enriched lncRNAs. A set of 684 lncRNAs had detectable expression (CEL-seq normalized count > 0 across the cell-type specific transcriptomes) (Table S1), 136 of which (~20%) were identified as being differentially expressed (*Q* < 0.05) between the three cell types (Tables S2–S5). Most of these lncRNAs are enriched in choanocytes or archeocytes (57% and 24%, respectively), with only five lncRNAs being pinacocyte-specific (Figure 1E).

We detected no significant structural differences between the 136 cell-type enriched lncRNAs and the remaining ubiquitously expressed lncRNAs (*n* = 548), with gene length (511 ± 36.28 vs. 451 ± 10.61 (average ± SEM), respectively; Mann-Whitney U test, *p*-value = 0.096244) and number of exons (1.9 ± 0.09 vs. 1.7 ± 0.05 (average ± SEM), respectively; Mann-Whitney U test, *p*-value = 0.086181) being similar. Moreover, these two groups of lncRNAs were not significantly different in relation to their positions and direction of transcription with respect to protein-coding genes (i.e., 49.6% vs. 49.2% intergenic co-location; and 20.2% vs. 17.7% having at least one exon that overlaps with an exon of a protein-coding gene on the opposite strand, respectively; Fisher’s exact test, *p*-value > 0.05 in both cases).

However, of the choanocyte-enriched lncRNAs, *AmqTCONS_00001337*, *AmqTCONS_00001338*, and *AmqTCONS_00001339* are in close genomic proximity to a cluster of immune-related genes (Tnf receptor-associated factors (TRAFs)) (Figure 1F), which are also differentially enriched in choanocytes [59]. Interestingly, the Tnf receptor-associated factor 4-like belonging to this cluster of immune-related genes (Aqu2.1.23792_001) was also co-expressed with these three lncRNAs (i.e., belonging to the same developmental co-expression module; see Table S7).

*Amphimedon* possesses nearly 300 genes from the scavenger receptor cysteine-rich domain-containing (SRCR) gene family, many of which are also differentially expressed in choanocytes; these are putatively involved in microbe-associated molecular patterns recognition [59,60,61]. These large complements suggest that this morphologically-simple animal without an apparent adaptive immune system could have the capacity to distinguish and subsequently generate specific responses to foreign and symbiotic bacteria [62]. Consistent with this premise, these three choanocyte-enriched lncRNAs were previously found to be co-expressed with protein-coding genes enriched for scavenger receptor activity [20] and up-regulated when *Amphimedon* juveniles were exposed to a foreign bacterial suspension belonging to a different sponge species (*Rhabdastrella globostellata*, Carter 1883) [59]. Together, these findings suggest a putative role for these three lncRNAs in innate immunity in *Amphimedon*.

Analysis of *AmqTCONS_00003141* cell-type expression profile revealed its upregulation in archeocytes and pinacocytes. This *cis*-antisense lncRNA is also co-expressed with protein-coding genes involved in key intercellular signaling pathways, including the G-protein-coupled receptor *Frizzled-B* (UniProt: I1G9T3_AMPQE) and *TGF-β* receptor type-1 (National Center for Biotechnology Information (NCBI) Reference Sequence: XP_011409575.1) [20]. Consistent with this, genes encoding *TGF-β*, a major immunosuppressive cytokine with a highly-conserved role in metazoan immunity [63,64] and development [65], are also differentially enriched in pinacocytes [59].

### 2.2. Sponge lncRNAs Show Cell Type-Specific Restricted Expression Patterns

**Figure 2:**
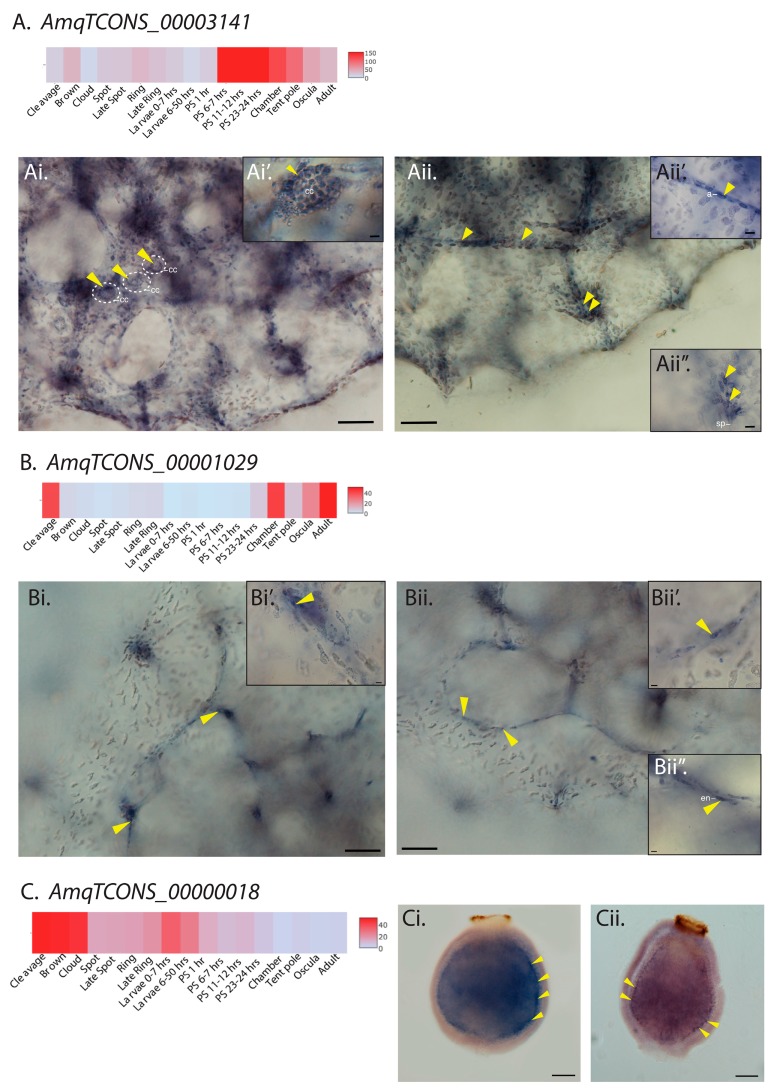
Cell type-specific restricted expression patterns of three candidate lncRNAs. (**A**) Heatmap representation of developmental expression of *cis*-antisense lncRNA *AmqTCONS_00003141*. (**Ai**,**Aii**) Whole mount in situ hybridization (ISH) of *AmqTCONS_00003141* to oscula stage juveniles; micrographs are views of the apical (top) side. Yellow arrowheads in (**Ai**,**Ai’**) show *AmqTCONS_00003141*-expressing choanocytes (cc) in chambers. Yellow arrowheads in (**Aii**) show clusters of AmqTCONS_00003141-expressing cells at the apex of tent-pole like structures, visible here as a vertically oriented cluster of spicules (sp) and associated cells (**Aii’’**), and aligned in streams running under the body surface. In both cases, these appear to be archeocytes (a) (**Aii’**). (**B**) Heatmap representation of developmental expression of lincRNA *AmqTCONS_00001029*. (**Bi**,**Bii**) Whole mount ISH of *AmqTCONS_00001029* to oscula stage juveniles; micrographs are views of the apical (top) side. Yellow arrowheads in (**Bi**,**Bi’**) indicate tent-pole like structures where there is strong expression of *AmqTCONS_00001029*. Yellow arrowheads in (**Bii**) indicate epithelial *AmqTCONS_00001029*-expressing endopinacocytes (en) (**Bii’**,**Bii’’**) that line the internal network of canals. (**C**) Heatmap representation of expression of the lincRNA *AmqTCONS_00000018*. (**Ci**,**Cii**) Whole mount ISH of larvae labeled with antisense riboprobe and viewed from the lateral side; anterior down. Yellow arrowheads show *AmqTCONS_00000018* expression in subepithelial cells at the boundary between outer cell layer and inner cell mass. Scale bars: 50 μm (**Ai**,**Aii**,**Bi**,**Bii**,**Ci**,**Cii**), 5 μm (insets in **Ai**,**Aii**,**Bi**,**Bii**).

To further validate the cell-type-specific expression of *Amphimedon* lncRNAs, we selected three independently regulated lncRNA transcripts with different developmental expression profiles for in situ hybridization (ISH) analysis; one upregulated in metamorphosing postlarvae (*AmqTCONS_00003141*), one upregulated in feeding 3-day old juveniles (*AmqTCON_00001029*) and one upregulated in larvae (*AmqTCONS_00000018*) (Figure 2).

*AmqTCONS_00003141* is activated 6–7 h after settlement and commencement of metamorphosis, and remains highly expressed as the juvenile body plan is forming (Figure 2A). Its transcripts were localized to subsets of specific internal cells of late postlarvae and juveniles (oscula stage), and not detected in the outer epithelial layer. Specifically, they were detected in a subset of choanocytes comprising newly-formed feeding chambers (Figure 2Ai,Ai’); ISH reveals only a fraction of choanocytes at these stages have detectable expression of *AmqTCONS_00003141*. Although highly expressed in choanocytes (i.e., belonging to quartile Q4; Table S6), this variability in expression might explain the absence of this transcript in the differentially expressed choanocyte-specific CEL-seq dataset (Table S5). At this stage, choanocytes chambers (as shown in Figure 2Ai,Ai’) typically contain proliferating cells and choanocytes from these chambers can rapidly dedifferentiate into archeocytes [66]. Consistent with some archeocytes being derived from dedifferentiating choanocytes, *AmqTCONS_00003141* transcripts were also detected in clusters of archeocytes, which are larger and often form migratory streams (Figure 2Aii,Aii’).

The previously characterized long intergenic ncRNA (lincRNA) *AmqTCONS_00001029* is a 526-nt transcript encoded by three exons, expressed from chamber (late postlarval) to adult stages [67] (Figure 2B). In contrast to *AmqTCONS_00003141*, its transcripts were detected in epithelial cells—endopinacocytes—that line the internal network of canals (Figure 2Bi,Bii’).

The remaining lincRNA (*AmqTCONS_00000018*), a 959-nt transcript encoded by two exons, was expressed in larval stages right before settlement (Figure 2C). *Amphimedon* larva has three cells layers; an outer epithelial layer interspersed with globular cells and flask cells, a subepithelial layer composed mostly of large globular, and the inner cell mass [68]. *AmqTCONS_00000018* transcripts were detected in subepithelial cells at the boundary between outer cell layer and inner cell mass (Figure 2Ci,Cii).

Together, as in bilaterians [19,21,22,69], *Amphimedon* lncRNAs are expressed in spatially- and cell type-restricted expression patterns, encompassing multiple cell types, consistent with lncRNA expression during animal development being highly dynamic [18,20] and tightly regulated to a specific developmental and cellular context. Although functional evidence currently is lacking, the restricted expression of these sponge lncRNAs in specific cell types during development, as observed in other animals [70], suggests that these non-coding genes are part of regulatory network(s) in *Amphimedon*.

### 2.3. Amphimedon and Sycon lncRNAs Are Co-Expressed with Similar Sets of Protein-Coding Genes

**Figure 3:**
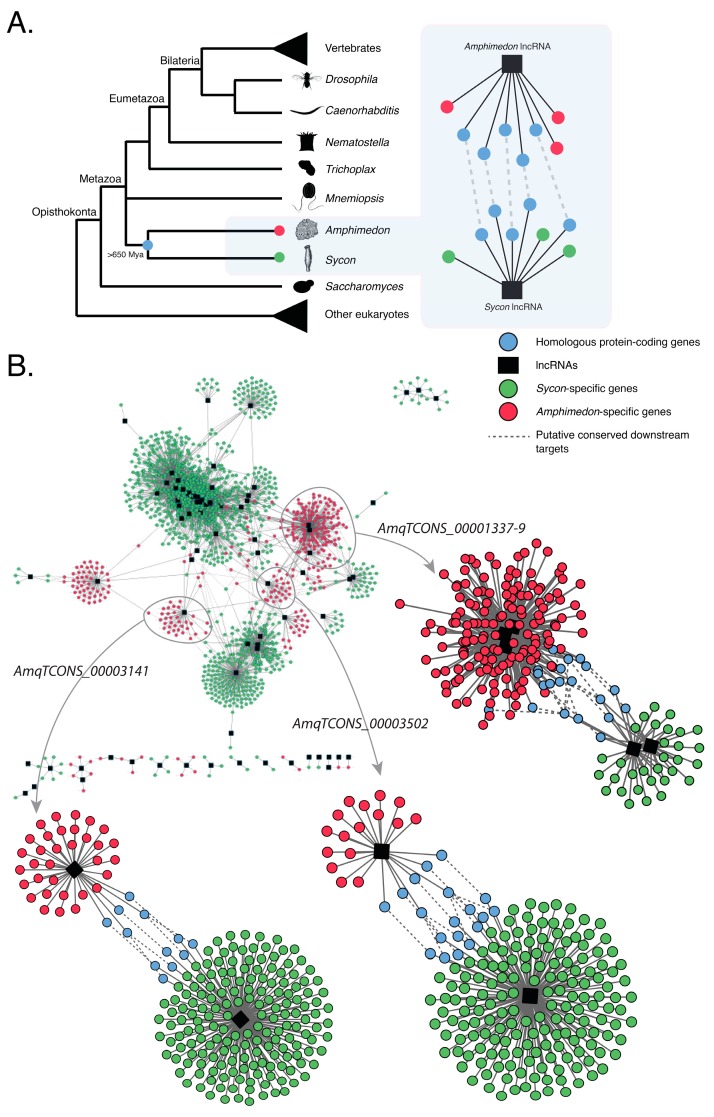
Putative evolutionarily conserved modules of co-expressed protein-coding genes and lncRNAs in the sponges *Amphimedon queenslandica* and *Sycon ciliatum*. (**A**) A conceptual model of how evolutionarily conserved networks of co-expressed homologous genes and lncRNAs can be inferred across divergent sponge lineages, despite the lack of lncRNA sequence conservation; (**B**) Co-expression networks based on differentially expressed protein-coding and lncRNA genes in *Amphimedon* [20] and *Sycon* [18]. Nodes indicate differentially expressed coding-genes, hubs (black) represent differentially expressed lncRNAs, and edges represent significant co-expression (both positive and negative). *Amphimedon*-specific genes are shown in red. *Sycon*-specific genes are shown in green. Conserved homologous genes shared between *Amphimedon* and *Sycon* are shown in blue. See Tables S7 and S8 for the complete edge and node lists of genes, and [20] for the developmental expression profiles of *AmqTCONS_1337-9*, *AmqTCONS_3502*, and *AmqTCONS_0003141* and their co-expressed protein-coding genes.

Previous findings have shown that *Amphimedon* and *Sycon* lncRNAs are co-expressed with similar sets of protein-coding genes [18,20], suggesting that, despite showing no apparent homology with any known animal lncRNAs, sponge lncRNAs may operate in evolutionarily conserved developmental modules (or networks) (Figure 3A).

To further document this correlation, we focused on the differentially expressed lncRNAs that strongly correlated with the expression profiles of sets of protein-coding genes involved in key animal developmental processes in the sponges *Amphimedon* and *Sycon* [18,20]. These two sponges are estimated to have diverged from each other at least 650 Mya [45]. We then constructed co-expression networks (modules), as a proxy for gene regulatory networks, based on these previously identified differentially expressed genes (both coding genes and lncRNAs) in both species [18,20]. These co-expression networks all have lncRNAs in central nodes, suggesting a key regulatory role for these lncRNAs (Figure S2).

Co-expression networks that consist of homologous protein-coding genes between *Amphimedon* and *Sycon* and differentially expressed lncRNAs were deemed to be conserved in sponges (Figure 3B). One such example is comprised of either developmental *Sycon* lncRNAs [18] or *Amphimedon* lncRNAs *AmqTCONS_1337-9*, *AmqTCONS_3502*, and *AmqTCONS_0003141* [20], which have no sequence similarity, and a similar set of homologous protein-coding genes (e.g., *TGF-β* receptor type-1) in both species (Figure 3B; Table 1; Tables S7 and S8).

The co-expression of lncRNAs with homologous coding genes in these sponges suggests these non-coding genes may operate in evolutionarily conserved co-expression networks. Alternatively, given there is no discernible sequence identity between these sponge lncRNAs and currently a lack of functional studies, it is also plausible that these putative co-regulatory networks have evolved independently in *Sycon* and *Amphimedon*, with lncRNAs being co-opted separately into homologous protein-coding networks.

## 3. Conclusions

The dynamic, cell type- and context-specific expression of sponge lncRNAs (this study; [18,20]) is consistent with spatiotemporal expression features of bilaterian lncRNAs also being present in sponges. The expression and possible function of lncRNAs during development can, therefore, be inferred to be present in the last common ancestor of these two lineages. Although currently there is a lack of functional data in sponges, lncRNAs appear to play a role in sponge development by regulating the deployment of various cell differentiation gene batteries as observed in bilaterians [70,71,72,73,74,75]. Given the lack of sequence identity of lncRNAs, it remains unclear if developmental sponge lncRNAs are conserved or independently-evolved. As gene regulatory networks and modules are central for the control and timing of animal development [76,77,78], the finding of similar sets of homologous protein-coding genes co-expressed and, thus possibly co-regulated, with lncRNAs between evolutionarily divergent sponge species, suggests lncRNAs may be playing important roles in these putative conserved gene regulatory networks.

## 4. Materials and Methods

### 4.1. Cell-Type Specific Transcriptome Analysis

A total of 39 samples were used from three adult *A. queenslandica* (5 archeocyte, 5 choanocyte and 3 pinacocyte samples from each adult individual). CEL-seq reads [58] from these samples were mapped back to the *A. queenslandica* genome [79] using Bowtie2 [80] and the CEL-seq analysis pipeline as previously described [81]. An average of ~9 million reads per sample were obtained, with an average of 60% of the reads mapped onto the *Amphimedon* genome [79]. All samples with less than 1 million reads formed their own cluster in the preliminary principal component analysis (PCA) using DESeq2 [82] and were therefore discarded from further analyses, resulting in a total of 31 samples (15 archeocyte, 10 choanocyte and 6 pinacocyte samples) used in this study (Table S1). The final counts were analyzed for differential gene expression using DESeq2 [82]. Pairwise comparisons were conducted between each of the three cell types to generate a list of differentially expressed genes for each cell type (Tables S2–S4). A 5% False Discovery Rate cut-off was used to produce the final lists of differentially expressed lncRNAs and protein-coding genes (Table S5). To investigate the full repertoire of lncRNAs expressed (in contrast with differentially expressed) in each cell type, the lncRNAs were also divided into expression quartiles. All the zero count reads were discarded and the median expression value of the non-transformed normalized count values of all samples (from all cell types) were used to calculate the quartile values. These values were used to classify the expression of all the lncRNAs in each cell type into four quartiles, ranging from low (Q1) to highly (Q4) expressed overall (Table S6).

### 4.2. Gene Isolation and Whole Mount In Situ Hybridization

*Amphimedon* lncRNA fragments were amplified with gene specific primers, by using complimentary DNA from mixed developmental stages as a polymerase chain reaction (PCR) template. Gene specific primers were as follows: AmqTCONS_00003141_Fw, ATAGGACCCACCCAGTCAAAC and AmqTCONS_00003141_Rev, TTCCTTGTTGTTCCTTGCCCT; AmqTCONS_00001029_Fw, AGA ATTGGCCGTAACAACAAGT and AmqTCONS_00001029_Rev, TCTAAGAAAATCTAAGTTACGTGTACG; AmqTCONS_00000018_Fw, TCCATTCCTATATTTTCCCCTTC and AmqTCONS_00000018_Rev, ATGAGGGTGGGATGATGTGC. The fragments were cloned into pGEM-T Easy (Promega, Fitchburg, Wisconsin, USA) vector using the manufacturer’s protocol and verified by sequencing using M13F and M13R primers. Digoxygenin (DIG)-labelled antisense RNA probes were transcribed from PCR products using DIG RNA Labeling Mix (Roche, Basel, Switzerland) and T7 or SP6 Polymerase (Promega, Fitchburg, Wisconsin, USA) following the manufacturer’s instructions. Whole mount ISH analysis of larval and juvenile gene expression was carried out as described previously [83]. Antisense DIG-labelled riboprobes were hybridized at a final concentration of 1 ng/μL. Whole-mount samples were observed under an Olympus SZX7 or a Nikon eclipse Ti microscope (Olympus Australia Pty Ltd., Mt Waverly, VIC, Australia) and photographed with a Nikon Sight DS-U1 camera (Nikon Australia Pty Ltd., Lidcombe, NSW, Australia).

### 4.3. Co-Expression Network Analysis

Co-expression networks were constructed based on the previously identified differentially expressed genes (coding genes and lncRNAs) in both *Amphimedon* [20] and *Sycon* [18]. Co-expression analysis in both species was performed as previously described (Gaiti et al., 2015). Co-expression networks were visualized using Cytoscape [84]. These networks show genes co-expressed with lncRNAs, where nodes indicate differentially expressed coding-genes, hubs indicate lncRNAs, and edges represent a significant co-expression (both positive ≥ 0.95 and negative ≤ −0.95) (*p*-value < 0.05). Homology between *Sycon* and *Amphimedon* was inferred with BLAST+ (version 2.2.30) [85], using BLASTp (*e*-value cutoff < 1 × 10^−5^) against a custom all vs. all database containing all *Amphimedon* Aqu2.1 peptides [86] and all peptides identified in the *Sycon* transcriptome [18] using TransDecoder (recommended settings, guided by UniProt and Pfam-A databases) [87]. Putative “evolutionarily conserved” modules were defined as modules containing at least one homologue between species.

### 4.4. Data Access

*Amphimedon* cell-type specific CEL-seq datasets can be obtained from NCBI under accession number PRJNA412708 [58]. *Amphimedon* genome assembly ampQue1 was used throughout the study. Developmental CEL-seq datasets used can be obtained from NCBI Gene Expression Omnibus (GEO) [88] under accession number GSE54364 [89]. The following gene model datasets were used for all analyses. *A. queenslandica*: Aqu2.1 models [86], lncRNAs [20]. *S. ciliatum*: coding genes and lncRNAs [18]. The codes used for the gene co-expression analysis are available for download at [20]. 

## Figures and Tables

**Table 1:** List of conserved homologous genes shared between *Amphimedon queenslandica* (Aqu2.1 prefix) and *Sycon ciliatum* (scigt prefix) for the three examples of putative evolutionarily conserved modules of co-expressed coding genes and lncRNAs. See Tables S7 and S8 for the complete edge and node lists of genes.

***AmqTCONS_00003141***	
**Homologous gene pairs**	**Description**
scigt010895-Aqu2.1.43387_001	mitochondrial dicarboxylate carrier
scigt017797-Aqu2.1.41074_001	protein disulfide-isomerase a5-like
scigt001771-Aqu2.1.30885_001	sh3 and px domain-containing protein 2a-like
scigt016036-Aqu2.1.36626_001	adp-ribosylation factor gtpase-activating protein 2-like
scigt018255-Aqu2.1.30885_001	sh3 and px domain-containing protein 2a-like
scigt000612-Aqu2.1.41568_001	tgf-beta receptor type-1
scigt008994-Aqu2.1.41568_001	tgf-beta receptor type-1
***AmqTCONS_00001337-9***	
**Homologous gene pairs**	**Description**
scigt017951-Aqu2.1.43947_001	arylsulfatase b-like
scigt017951-Aqu2.1.24502_001	arylsulfatase b-like
scigt017951-Aqu2.1.39727_001	arylsulfatase
scigt017951-Aqu2.1.41029_001	arylsulfatase
scigt017951-Aqu2.1.37909_001	sulfatase
scigt014545-Aqu2.1.37909_001	sulfatase
scigt014545-Aqu2.1.41029_001	arylsulfatase
scigt014545-Aqu2.1.39727_001	arylsulfatase
scigt017997-Aqu2.1.32274_001	usherin
scigt020120-Aqu2.1.28087_001	lysosomal alpha-glucosidase-like isoform x2
scigt020423-Aqu2.1.35119_001	filamin-c-like isoform x3
scigt000557-Aqu2.1.32241_001	myosin-i heavy chain
scigt008273-Aqu2.1.36394_001	deleted in malignant brain tumors 1
scigt017951-Aqu2.1.42755_001	arylsulfatase b-like
***AmqTCONS_00003502***	
**Homologous gene pairs**	**Description**
scigt000138-Aqu2.1.44676_001	actin family protein
scigt001771-Aqu2.1.38758_001	tyrosine-protein kinase lck
scigt005362-Aqu2.1.44676_001	actin family protein
scigt004922-Aqu2.1.40987_001	unconventional myosin-viia
scigt008792-Aqu2.1.24982_001	adenylyl cyclase-associated protein 1
scigt012572-Aqu2.1.40987_001	unconventional myosin-viia
scigt014349-Aqu2.1.32914_001	pleckstrin homology domain-containing family g member 1-like
scigt016045-Aqu2.1.28519_001	ap-2 complex subunit alpha-1-like
scigt020995-Aqu2.1.43989_001	protein plant cadmium resistance 3-like
scigt021992-Aqu2.1.44676_001	actin family protein
scigt022018-Aqu2.1.44676_001	actin family protein
scigt025009-Aqu2.1.40987_001	unconventional myosin-viia

## Supplementary Materials

The following are available online at http://www.mdpi.com/2311-553X/4/1/6/s1, Figure S1: Schematic representation of the evolutionary relationship among representative sponge species. Blue box highlights the sponge clade. *Amphimedon queenslandica* and *Sycon ciliatum*, the sponge species used in this study, are shown in bold red. For detailed phylogenetic relationship analyses of sponges please refer to [46,47,48,52]. Adapted from [20]. Figure S2: Co-expression networks of *Amphimedon* and *Sycon* lncRNAs. Co-expression networks are based on the previously identified differentially expressed genes (coding genes and lncRNAs) in *Amphimedon* [20] and *Sycon* [18]. Nodes indicate coding-genes, hubs (black) represent lncRNAs, and edges represent significant co-expression (both positive and negative). Table S1: Cell-type specific CEL-seq normalized read counts of *Amphimedon* lncRNAs and protein-coding genes across all samples. Table S2: Differential gene expression of *Amphimedon* lncRNAs and protein-coding genes between archeocyte and choanocyte. Only genes that passed our technical cut-off of a 5% False Discovery Rate (FDR) are shown. Table S3: Differential gene expression of *Amphimedon* lncRNAs and protein-coding genes between archeocyte and pinacocyte. Only genes that passed our technical cut-off of a 5% False Discovery Rate (FDR) are shown. Table S4: Differential gene expression of *Amphimedon* lncRNAs and protein-coding genes between choanocyte and pinacocyte. Only genes that passed our technical cut-off of a 5% False Discovery Rate (FDR) are shown. Table S5: Final lists of differentially expressed *Amphimedon* lncRNAs and protein-coding genes for each cell type at a 5% FDR (*Q* < 0.05). The examples provided in the main text are highlighted in yellow. Table S6: The number of lncRNAs expressed in each cell type for each quarter and combined quarters, using expression quartile values (Q1: 1.45, Q2: 1.95, Q3: 3.11). Table S7: Node tables for the three specific examples of evolutionarily conserved modules of co-expressed coding genes and lncRNAs between sponges shown in Figure 3. Table S8: Edge tables for the three specific examples of evolutionarily conserved modules of co-expressed coding genes and lncRNAs between sponges shown in Figure 3. Nodes are marked as ‘name’ and edges are listed as ‘interaction’.
