# Supplementary figures and images for: Sponge Long Non-Coding RNAs Are Expressed in Specific Cell Types and Conserved Networks
Source: Noncoding RNA. 2018 Mar 7;4(1):6. doi: 10.3390/ncrna4010006 (PMC5890393; doi:10.3390/ncrna4010006)

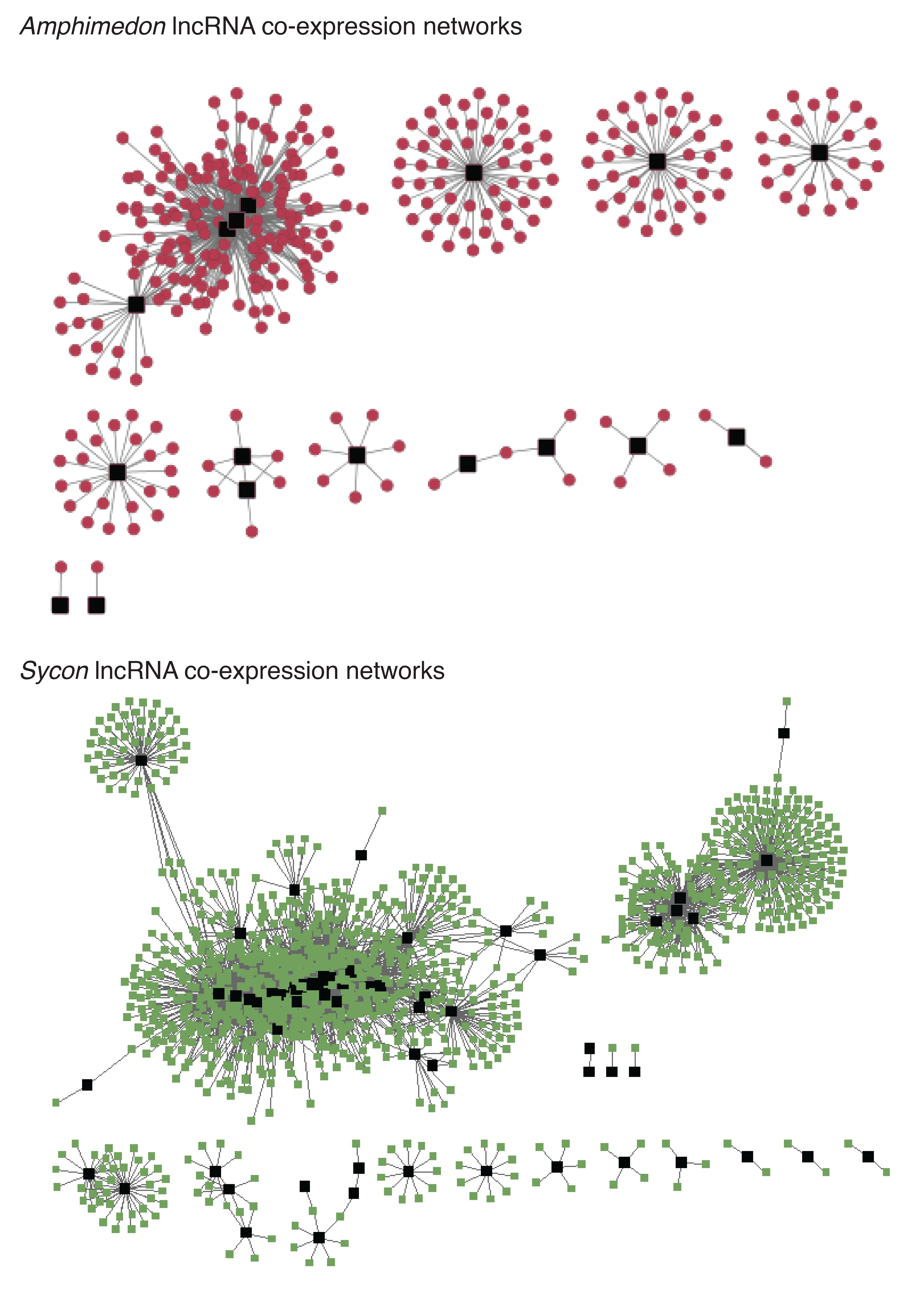

Supplement: Supplementary file 1 [file ncrna-04-00006-s001.zip › Supp Files/Gaiti et al_Fig.S2.tif]

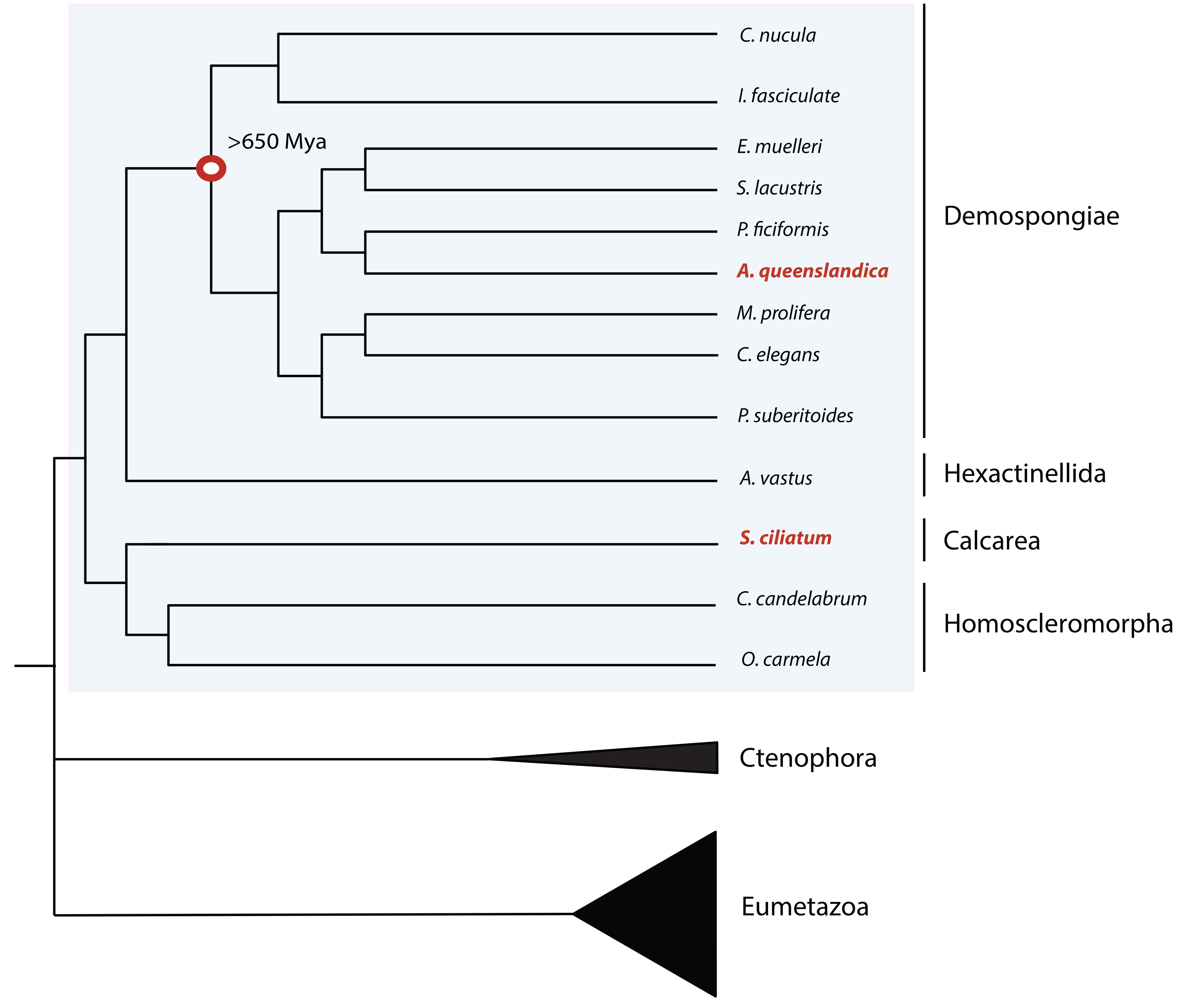

Supplement: Supplementary file 1 [file ncrna-04-00006-s001.zip › Supp Files/Gaiti et al_Fig.S1.tif]
